# Chemomimesis and Molecular Darwinism in Action: From Abiotic Generation of Nucleobases to Nucleosides and RNA

**DOI:** 10.3390/life8020024

**Published:** 2018-06-20

**Authors:** Raffaele Saladino, Judit E. Šponer, Jiří Šponer, Giovanna Costanzo, Samanta Pino, Ernesto Di Mauro

**Affiliations:** 1 Biological and Ecological Department, University of Tuscia, 01100 Viterbo, Italy; saladino@unitus.it (R.S.); samantapino78@libero.it (S.P.); 2 Institute of Biophysics of the Czech Academy of Sciences, Královopolská 135, 61265 Brno, Czech Republic; sponer@ncbr.muni.cz; 3 Institute of Molecular Biology and Pathology, CNR, 00185 Rome, Italy; giovanna.costanzo@uniroma1.it

**Keywords:** origin of life, systems chemistry, Chemomimesis, Molecular Darwinism

## Abstract

Molecular Darwinian evolution is an intrinsic property of reacting pools of molecules resulting in the adaptation of the system to changing conditions. It has no a priori aim. From the point of view of the origin of life, Darwinian selection behavior, when spontaneously emerging in the ensembles of molecules composing prebiotic pools, initiates subsequent evolution of increasingly complex and *innovative* chemical information. On the *conservation* side, it is a posteriori observed that numerous biological processes are based on prebiotically promptly made compounds, as proposed by the concept of Chemomimesis. Molecular Darwinian evolution and Chemomimesis are principles acting in balanced cooperation in the frame of Systems Chemistry. The one-pot synthesis of nucleosides in radical chemistry conditions is possibly a telling example of the operation of these principles. Other indications of similar cases of molecular evolution can be found among biogenic processes.

## 1. Introduction

In the absence of life, the components of biogenic processes were necessarily generated in abiotic reactions [1,2,3,4,5]. The conditions under which these syntheses occurred and may still occur are multiform and, as such, are widespread in the Universe. Hence the observations in different interstellar spaces and in different lifeless celestial bodies of molecules which, on our Planet, are starting points and/or are part of biological systems [6,7,8,9,10].

The chemical composition and complexity of the pools of potentially biogenic compounds differ, necessarily depending on a large number of parameters. Many of these parameters are still poorly characterized or are possibly unknown. Nevertheless, it is increasingly clear that prebiotic syntheses occur under a variety of energy sources, of different mixtures of simple starting compounds, of catalysts, and of physico-chemical conditions. Is it possible to identify some of the principles guiding their evolution towards Life?

## 2. The Principles Underlying Progress towards Further Complexity

Darwinian selection has no aims; it does not work for a purpose. It only has consequences, the major of which being the adaptive variation, otherwise called “evolution”, of the system following the modification of the conditions in which the system has existed thus far. The process of “adaptive variation” implies “adaptation” to the new conditions. The word “adaptation” describes the qualitative/quantitative modification of the components of the system as a consequence of the process started by the variation of the initial conditions.

When dealing with a population of molecules generated in a synthetic system endowed with biogenic potential, the variation of the conditions of the system depends upon external and internal factors. Internal factors essentially consist of the singly independent and/or of the multiple interacting reactivity of the molecules present. In the absence of special quenching factors, all the molecular populations produced in prebiotic synthetic pools are bound to evolve up to a given point, adapting themselves to the environment that their synthesis has contributed to establish, till exhaustion of the intrinsic reactivity of the system. As discussed below, energy aspects are paramount. What could be hinted at by recent findings on prebiotic synthetic pools about prebiotic evolutionary processes?

## 3. Principles for Systems Chemistry

One way of considering the progress of first-generation prebiotic pools towards biogenic processes is to consider them at the light of Systems Chemistry. In Systems Chemistry [11,12,13,14] the focus does not a priori lie on individual chemical components, but rather on the overall ensemble of interacting molecules and on their emergent properties. Systems Chemistry would benefit from the definition of working principles. We propose to use the expression “Systems Chemistry” for the ensemble of considerations dictated by Molecular Darwinism and Chemomimesis.

Molecular Darwinism is a term first introduced, to the best of our knowledge, by J. S. Wicken [15], who critically considered it as a primordial selective process based on unwarranted assumptions. The term was later progressively used as a means to refer to genetic phenomena at the molecular level, like the principle underlying spontaneously occurring genetic variants as driving force of biological evolution by W. Arber [16]. The complexity level considered was high: local sequence changes, intragenomic reshuffling of DNA segments, acquisition of a segment of a foreign DNA, and the like. This complexity defines the purport of the term at a mature biological level. In what follows, we use this term in the meaning originally suggested by the Göttingen school of Molecular Darwinism, which extends the operation of Darwinian principles of random mutations and selection to chemical processes occurring at the abiotic level of complexity [17]. Molecular Darwinism is Chemical Evolution in Higgs’ purport [18], with the additional attributes of intrinsic selection and competition processes which is the core essence of Darwinism.

Chemomimesis is a term introduced by A. Eschenmoser and E. Loewenthal [19] to indicate that chemical compounds and processes characterizing biological phenomena often have purely abiotic precedents: something is copied and used that already existed. In trying to understand the mechanisms characterizing the passages from the abiotic through the prebiotic to the biotic, Chemomimesis is a powerful concept [20,21] which, however, can only be applied according to a posteriori logics: a natural process becomes chemomimetic *after* the organisms which use it have come into being. The combination of Molecular Darwinism and Chemomimesis may be instrumental for a fact-based understanding of the abiotic-to-prebiotic-to-biotic paths.

## 4. One-Pot Initial Events under a Variety of Energy Sources: An Example

Pools of potentially prebiotic compounds are obtained in early-Earth conditions [22,23], in hydrothermal environments [24,25], and in irradiated and/or impacted Earth atmosphere [26,27]. The HCN and formamide (NH_2_COH) chemistries are interrelated [28,29] and are the natural chemical frames into which a rich panel of prebiotic compounds have been obtained. The ubiquitous [30,31,32,33] compound formamide has in particular shown its worth [34,35,36,37], due to its peculiar physico-chemical properties [34,35], allowing its liquid state to have a 200 °C-wide interval, as well as its facile accumulation [38,39]. In Ref. [40], we suggested a prebiotic scenario, which assumed that liquid formamide could accumulate on some hot surface on the early Earth at temperatures around 180 °C as a thermal dissociation product of ammonium formate. This paper responds to the critical notes of Bada et al. [41], who demonstrated that at room temperature formamide is highly hygroscopic, i.e., in these conditions it could not accumulate in a concentrated form. We have been pleased to learn that in a recent report, one of the authors of Ref. [41] has changed his mind and has experimentally demonstrated that far above the boiling point of water (in line with our proposal in Ref. [40]) formamide can be accumulated in concentrated form [42].

Noteworthily, formamide was shown to be the key intermediate in the Urey-Miller reactions [43]. It has been proposed that among all the chemical scenarios tested so far, formamide is a favored starting compound as far as the complexity of the resulting mixtures of products is concerned [34,35]. Formamide is also remarkable for its versatility: its synthetic capacities are evident in all the physico-chemical environments tested and under the effect of a great variety of catalysts [34,35,44,45,46,47,48,49,50,51]. As for the energy source triggering the formamide-based prebiotic syntheses, large panels of products were observed under heat, UV, ionizing and proton irradiation, as reviewed [9].

**Figure 1:**
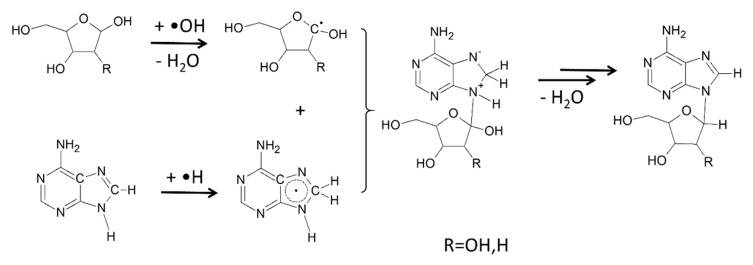
Proposed mechanism of the proton irradiation induced *N*-glycosidation between adenine and ribose [69].

The idea that prebiotic pathways were straight, streamlined and fastidiously demanding (see Figure 1, in Ref. [37]) is contradicted by the promiscuous efficiency of formamide-based comprehensive syntheses. We suggest that a combination of external conditions, for example, exposing the system simultaneously to variable proton irradiation, UV irradiation and temperature conditions may lead to further complexification of the chemical composition, as the individual external factors may act in synergy. A physicist would suggest that combining variations in several external factors in a combinatorial way may create a “multidimensional response” in the resulting composition of the chemical system, driving the system through diverse “chemical trajectories” on the “chemical compositional landscape”. Such scenarios are certainly not irrelevant in the context of the prebiotic Earth, considering the time and size scale available for the onset of chemical evolution.

Formamide-based syntheses carried out under proton irradiation yielded the structurally most complex set of compounds obtained in one-pot reactions [46], including the sugars ribose and 2′-deoxyribose, and the canonical nucleobases (cytosine, uracil, adenine, guanine, and thymine). Most notably in the prebiotic perspective, the four nucleosides uridine, cytidine, adenosine, and thymidine were also synthesized. In this latter study, proton irradiation of formamide was performed in highly controlled conditions using 170 MeV proton beams generated by accelerated Helium at the Phasotron facility, Joint Institute for Nuclear Research, Dubna, Russia. In these conditions the prevailing chemical scenario is a bona fide radical chemistry, implying participation of cyano radical species (•CN).

Radical chemistry may occur in different environments and may be triggered by different causes, as reactions at very low temperatures, break-up of larger molecules, heat, electrical discharges, electrolysis or, in particular, ionizing radiations. One remarkable property of radical chemistry is that the presence of unpaired electrons makes free radicals highly reactive both towards neutral molecules as well as towards themselves. As a consequence, radical chemistry may be particularly relevant when dealing with dimerizations and polymerizations. If a reactant has been activated by, say, irradiation, its activated radical state could allow its further reactions to occur in conditions in which closed-shell molecules could not react (i.e., at lower temperatures). Hence, radical chemistry is the favored system for space and/atmospheric prebiotic chemistry studies and could allow reactions to proceed through intermediate stages which are not accessible to non-radical compounds. An example is provided by the above-mentioned one-pot condensation of formamide up to nucleosides [46], accompanied in the same reaction pool by the synthesis of other different types of molecules, from amino acids and carboxylic acids, to molecules as complex as C18 and C20-compounds, like stearic acid and arachidic acid. In addition to the interest of the production of chemical information-bearing molecules per se, the prebiotic relevance of radical chemistry-based scenarios is enhanced by the overall increased complexity of the pool of the afforded compounds. Radical chemistry-related synthesis of nucleic acid bases was also reported in other studies in different conditions [27,52,53].

As Benner noted, prebiotic chemistry without selection leads to tar formation [54,55]. As several literature examples on the oligomerization of HCN [56,57] demonstrate, this statement is especially true for radical chemistry. A possible way to overcome tar formation is binding to minerals [58]. As demonstrated in Refs. [27,46], formamide-based radical chemistry combined with catalysis by meteorites could provide a plausible solution for this problem.

## 5. From Complex Mixtures to Pre-Genetic Materials

A reproducible transmission of genotype is the consensual essence of “Life” [59,60]. It could only have started through the fertile interaction of pre-genetic materials with metabolism-wise energy control and membrane-based containment devices. The pools of molecules obtained in one-pot syntheses from formamide encompass compounds relevant for each of these three independent to-be-converged domains. Recent progress has been marked more for pre-genetics and for bio-vesicles [61,62,63] than for pre-metabolisms. Focusing on pre-genetics, new data and new scenarios are currently being proposed. Reports abound on the abiotic syntheses of nucleobases and of nucleosides, on the mechanisms for their possible phosphorylation, on their oligomerizations, and on the properties which endow them with possible selective evolutionary advantages, as we describe below.

## 6. The Nucleobases are the Right Ones since the Beginning. The Case of AICA and fAICA

Adenine and guanine are the pivotal compounds for genetics (the genotype) and for metabolism (the phenotype). Protein synthesis, which connects the two, is not conceivable without ATP or GTP. The imidazoles AICA (4-aminoimidazole-5-carboxamide) and AICAI (4-aminoimidazole-5-carboxamidine) are the relevant intermediates in the chemical synthesis of purines, as first described for the synthesis of adenine from a concentrated solution of ammonia and HCN [64]. AICA and fAICA (5-formamidoimidazole-4-carboxamide) are intermediates of the biosynthesis of inosine-5′-monophosphate (IMP), the main route to purine nucleotides in extant cells. The similarity between the intermediates of this metabolic process and the chemical route described by Oró even increases when considering the compounds obtained in the condensation of formamide into adenine and hypoxanthine (which is the stable version of guanine, because it lacks the labile NH_2_ group in C-4 position of the purine ring) when reacted in the presence of a variety of catalysts and under different energy sources.

Similarly, in the frame of formamide chemistry, six of the eight carboxylic acids which are intermediates of the extant Krebs cycle have been detected under UV irradiation in the presence of titanium dioxide, highlighting the possibility of the total synthesis of a large part of the chemical machinery utilized by one of the cell’s oldest metabolic pathways [65]. The robustness of this chemical pathway is further evidenced by the formation of Krebs cycle intermediates from formamide under a variety of prebiotic scenarios, including iron-sulfur minerals [66], borates [67], zirconium minerals [68], and meteorites [45].

These observations provide a clear indication of the operation of Chemomimesis for compounds which are central to both genetics and energy control, apparently starting from the very beginning.

## 7. Focusing on Nucleosides

Performing, in the same conditions as those used for their synthesis [46], proton irradiation on mixtures of preformed sugars and adenine in the presence of a chondrite meteorite allowed the analysis of the reaction leading to the formation of the *β*-glycosidic bond [69]. These conditions simulate the presumptive conditions in space or on an early Earth fluxed by slow protons from the solar wind, sketching a potentially prebiotic scenario. The reaction consists of the formation of the *β*-glycosidic bond between separately preformed sugar and nucleobase moieties (both of which can be prebiotically obtained in the same reacting pool, as described in Ref. [46]), thus providing a simple alternative to the complex pathways suggested for the prebiotic formation of nucleosides. These latter ones are based on the involvement of oxazoline chemistry [70] in the synthesis of pyrimidine nucleosides [71,72,73], and on the synthesis of purine nucleosides through the formamido-pyrimidines (FPy) chemistry [74]. The point on these approaches was recently made [75].

The possibility of studying the formation of nucleosides in one-pot reactions makes possible the analysis of the factors that might have played a role in the condensation of nucleotides into polymers, eventually leading to the evolution of extant nucleic acids. The analysis of stereoselectivity, regioselectivity, and the possibility of (poly)glycosylation of the nucleosides formed in this reaction set was, in particular, made possible [69].

The relevance of this detailed information resides in the fact that extant RNA is built based on a structure consisting of phosphodiester bonds formed along a sequence of strictly stereo- and regioselective nucleosides. DNA has conserved these selectivities. Thus, a selection was exerted on the pool of sugars potentially formed in the synthetic first ur-reactions, eventually leading to the phenotype of the polymeric molecule that resulted in being the most adaptable to self-reproduction and to codogenic roles. In the absence of any finalism, the selection was necessarily initially based on the most basic phenotypes: reciprocal structural affinity of the precursors, energetic compatibility in the polymerization process, survival capacity of the resulting polymer. Stabilization of the phosphorylated precursors may be acquired through several mechanisms, important among which is the cyclization of the phosphate moiety and the self-protection through polymerization [76,77,78] (see below). Survival of the polymer mostly depended on resilience towards hydrolysis and other degradative reactions, thus entailing its possible accumulation.

## 8. Regio- and Stereoselectivity of Nucleoside Formation is Conserved from the Beginning

In the radical chemistry-based proton irradiation-powered one-pot reaction between adenine and 2-deoxyribose (for a summary of the mechanism, see Figure 1), the formation of mono- and poly-glycosylated nucleosides was observed, affording: *α*-d-2′-deoxy-ribofuranosyl adenine, *β*-d-2′-deoxy-ribofuranosyl adenine, *α*-d-2′-deoxy-ribopyranosyl adenine, and *β*-d-2′-deoxy-ribopyranosyl adenine. Poly-glycosylated *N***^6^**-2′-deoxy-ribofuranosyl- and *N***^6^**-2′-deoxy-ribopyranosyl-2′-deoxyadenosine isomers were detected, and higher molecular weight poly-glycosylated derivatives, corresponding to the addition of up to six sugar moieties, were also observed [69].

The reaction of adenine with ribose afforded *α*-d-ribofuranosyl adenine, *β*-d-ribofuranosyl adenine, *α*-d-ribopyranosyl adenine, and *β*-d-2′-deoxy-ribopyranosyl adenine. Furanosides are the anomeric form present in extant nucleic acids. 2′-Deoxyribonucleosides formed more efficiently than ribonucleosides, and the *β*-isomer prevailed over the *α*-isomer.

As for the nucleobase regioselectivity of the glycosylation, the reaction selectively afforded N9 isomers, which are the isomers that molecular evolution has selected for the formation of nucleic acids. A mechanistic explanation was given for the absence of glycosylation on N1 and N7 of adenine [69].

These observations point to the fact that at least one reaction system exists [69] which allows the one-pot synthesis of the right components right from the beginning. Here, Darwinism worked on the evolution of new functions and Chemomimesis maintained the chemical structures.

## 9. Chemomimetic RNA

Artificial nucleic acids may exist in a large number of chemical alternatives [79,80,81,82]. The exploration of all the alternative possibilities is limited only by the ingenuity of the chemist. On the other hand, biological RNA and DNA are universal, unique, and very conserved. All the existing biological variants are epigenetic modifications of an evolutionarily unaltered chemical blueprint. If the initial pool had a nucleotide composition similar to the one that we have just described, we could a posteriori reason that RNA evolved to be composed of N9 isomers and of furanosides just because these forms were present as major species already in Darwin’s “*warm little pond*”. This is largely an example of Chemomimesis. Furthermore, polymers built as RNA and DNA are built (on that very backbone and using those very furanosides to which N9 isomers are bound) have the balanced properties of (i) stability, (ii) possibility of replication, and (iii) codogenicity, which makes them the best fit to fulfill the multiple roles that genetics needs from them. These properties were acquired, certified, and maintained through Darwinian Molecular evolution.

## 10. Exploring the Environment for the First Effective Phosphorylation Agents

The reasons why nature chose phosphate as the link to hold and maneuver genetic information have been conclusively reviewed [83]. The sources of phosphate for the prebiotic phosphorylation of nucleosides have long been debated [84,85,86,87], and the topic has been surrounded by a reasoned skepticism about the possibility of ever knowing them [88,89]. A. M. Schoffsthal reported in four studies between 1976 and 1988 the phosphorylation of nucleosides in the presence of formamide [90,91,92,93] from soluble phosphates and, lastly, from hydroxylapatite. Extending these studies [94,95], it was shown that nucleosides could be phosphorylated in the presence of many different phosphate minerals, provided the presence of a dissolving agent and of high temperature (≥400 K). Formamide efficiently fulfills this latter role, as does (less efficiently, and/or requiring longer times) water. The time scale of the mineral world may well be different from that of biology. Especially hydroxylapatite was shown to be a good source of phosphate for nucleoside phosphorylation. In this reaction, phosphorylation occurs at every possible position of the sugar moiety, at 2′, or at 3′, or at 5′ [94]. With time, the open forms (2′-, or 3′-, or 5′-XMPs) are degraded, while the more stable cyclic forms remain. These can be 2′, 3′ or 3′, 5′ cyclic XMPs. Chemically related solvent systems, based on urea as originally proposed in L. Orgel’s studies [86,87], have also recently been shown to be effective [96]. In addition, phosphorylation under aqueous conditions may occur from diamidophosphate, a compound derived from trimetaphosphate [97], whose prebiotic plausibility is claimed ibidem.

In conclusion, the phosphorylation of nucleosides may occur from numerous sources of phosphates and under a variety of conditions [98]. Which source [99] and which condition, among the various possibilities, was actually frequented in the *warm little pond* depends on their coherence with the steps that followed on the evolutionary path. From the point of view of Chemomimesis, the logic is clear: phosphorylation spontaneously occurs if the mineral environment, the solvent and the temperature are the right ones. The RNA structure a posteriori tells us that the process has been chemomimetically adopted and copied over. From the point of view of Molecular Darwinism, it all depends on the phenotype considered.

## 11. Focusing on Differential Kinetic Stability

In terms of possibility to evolve, the key molecular phenotype of any compound, both pre-biological and biological, is *stability*. Stability may be of kinetic or of thermodynamic nature. Among the two, thermodynamic stability is more universal (as Clemens Richert once said, “one can never fool thermodynamics”), whereas kinetic stability may be easily tuned with catalysts/inhibitors. At lower levels of chemical complexity (synthesis of prebiotic building blocks [27,69]), thermodynamic stability plays a more decisive role, while kinetic stability dominates at the level of biological molecules. 

Metabolic cycles in modern organisms, which are based on non-equilibrium chemistry, are kept alive due to kinetic barriers. Indeed, enhancement of the kinetic stability of nucleosides and nucleotides could drive Molecular Darwinism towards oligonucleotide sequences. The stability of the components of nucleic acids was analyzed in the 1960s and the 1970s under various physico-chemical conditions. Several differences were determined: (i) the rate of cleavage of the glycosidic bonds of free deoxynucleosides [100,101] is 10–50 times higher relative to that in single-stranded DNA [102]; (ii) the rate of hydrolysis of glycosidic bonds varies in the order deoxynucleosides > deoxynucleotides > DNA [103,104,105]; (iii) the depurination is 4-fold in single- versus double-stranded DNA (rate constant of single-stranded DNA = 4 × 10^−9^ s^−1^, 70 °C, pH 7.4) [106]. As a trend, higher molecular complexity allows higher stability. The stability of the phosphoester bonds determined in early studies has been reviewed [107]. A systematic comparison of the stability of the phosphoester bonds in precursor monomers (both ribo- and 2′-deoxyribo-) and that in DNA or RNA [76,77], has shown that the stability of the phosphoester bonds strongly depends on the molecular structure in which it is embedded, as well as on the solvent environment. In particular, the 3′ phosphoester bond (the fragile and active site of the RNA molecule) is more stable towards hydrolysis when incorporated in RNA than in monomers, both in water and at high concentrations of formamide [77]. The higher stability of the polymeric form establishes a basically important evolutionary advantage: longer survival. Interestingly, and expectedly, different RNA sequences have different stabilities [78], thus being endowed with different fitness. In this respect, exploration of sequence space corresponds to exploration of safer thermodynamical niches.

## 12. Focusing on Oligomerization

**Figure 2:**
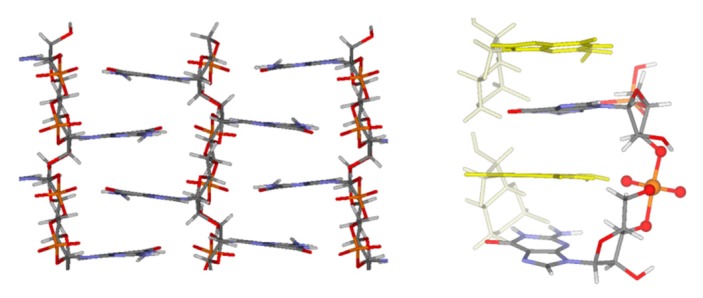
A ladder-like stacked supramolecular architecture provides optimum steric conditions for the oligomerization of 3′, 5′ cGMP. Left: Nucleobase stacking in the crystal structure of 3′, 5′ cGMP [119]. Right: Proposed structure of the trigonal bipyramidal intermediate of the chain-extension reaction from TPSS-D2/TVZP calculations [114]. The yellow nucleotides serve as mediators of the transphosphorylation reactions.

Attention was devoted in the 1970s to the cyclic forms of nucleotides as potential actors of abiotic polymerization [108,109] and as effectors of nucleic acids ligation and stability [110,111]. Studies on the possible origin of RNA self-polymerization from 3′, 5′ cyclic monophosphates were later resumed, showing that self-oligomerization may occur efficiently for 3′, 5′ cGMP [112,113,114,115], and less so for 3′, 5′ cAMP [116] and 3′, 5′ cCMP [117]. Thermodynamic arguments suggested the preferential accumulation of 3′, 5′ cyclic nucleotides over the 2′, 3′ isomers in a formamide-rich environment [118]. Among the four 3′, 5′ cyclic nucleotides, the markedly higher observed oligomerization efficiency of 3′, 5′ cGMP was explained by the unique self-assembling properties of this molecule [114]. It was shown that in this particular case a special stacked supramolecular architecture formed which provided optimum steric conditions for an anionic ring-opening living polymerization mechanism (see Figure 2). Thus, the favorable entropic factor ensured a kinetic fitness for the oligomerization reaction and for Molecular Darwinism to give rise to the emergence of oligoG sequences. 

Non-enzymatic polymerization of RNA precursors occurs in several systems other than cyclic nucleotides. The efficient polymerization of highly activated precursor monomers (usually phosphorimidazolides) has been explored [120,121,122], the results providing important information on in vitro evolutionary behaviors of RNA populations and on their interactions with other systems, i.e., membranes [123]. The relevance of phosphorimidazolides or even of triphosphate nucleotides in early prebiotic scenarios has been, however, questioned [108,124], due to their elaborate synthesis and high energy content, resulting in their difficult accumulation and prebiotic availability. Shortly, from the point of view of Molecular Darwinism, activation by imidazole breaks the systematic trend outlined by the energetics of the so far demonstrated synthesis of nucleic acid building blocks, which always progresses towards increasing stability. A reaction system allowing copying of RNA sequences based on local and transient formation of phosphorimidazolides was described [125]. The polymerization of acyclic monophosphate nucleosides in acidic conditions has been reported [126,127]. From an energetic point of view this process is compatible with the concept of Molecular Darwinism: in this case, binding of a proton/positively charged cation to the phosphate moiety of the nucleotides [128] combined with the formation of a stacked and H-bonded supramolecular architecture ensures increased kinetic fitness to the transphosphorylation reactions that lead to oligonucleotide formation. 

## 13. Chemomimesis of Cyclic Nucleotides

Cyclic nucleotides provide a striking example of functional plasticity in compounds that can be adapted to multiple uses while remaining in the genetic domain. The facile prebiotic phosphorylation of nucleosides [94], their higher stability as cyclic structures [94], the higher thermodynamic stability of the 3′, 5′ forms in formamide [118], all point to their presence in the *warm little pond* since the beginning, endowed with dedicated functions. As demonstrated [114], oligomerization of 3′, 5′ cyclic nucleotides offers a plausible way to overcome the water-paradox [4] that principally hampers oligonucleotide formation from acyclic nucleoside phosphate precursors in an aqueous environment.

Chemomimesis is considered to be a principle valid both for monomeric molecules and for processes. Nothing prevents the extension of its validity also to polymers. Given the spontaneous polymerizations reported for cyclic nucleotides, for preactivated phosphoimidazolides, and for monophosphates, it seems reasonable to assume that, one way or another, RNA prebiotically generated itself. Thus, extant biological RNA itself is a product of Chemomimesis. For RNA, mimesis is valid for the overall structure since the very beginning, while evolution pertains to the functions that it acquired along the way.

## 14. Concluding Remarks

Proto-life resulted from processes which were not programmed a priori, undergoing molecular adaptations dictated by the compositions of the prebiotic molecular pools, by the properties of the constituent molecules, by the environment, and by the history of the system. The principles at the basis of this selection processes are largely dictated by the quality/quantity of the compounds present, which results from both the first-run synthetic events and from their further-generation reactions, and is strongly influenced by the energetics of the system. A marked initial complexity is instrumental in determining the evolution of additionally complex chemical systems.

The rich and variegated pools of compounds, encompassing from nucleobases to nucleosides, generated by formamide chemistry in conditions allowing their further reactions and development of complexity, are a possible example of a Darwin’s *warm little pond*. Up to what point does the initial reactivity generate pre-genetic complexity? Somehow, to our surprise, we observed that the one-carbon atom formamide system may go a long way, especially so in radical chemistry conditions.

In complex systems, selection functions based on the most adapted, not on the most abundant. “*Adapted to what?*” can be rephrased into: which are the relevant phenotypes for selection in the absence of an established and functioning biological apparatus?

Among the properties that may drive the system towards complexity, creating the selective conditions for further interactions and higher complexity, one should consider *thermodynamic stability*. Another important parameter is the *kinetics of the reactions* because it determines which reaction occurs preferentially before the final steady-state of the reacting pool is reached. Kinetics and thermodynamics are both influenced by *entropic factors*, which is the manifestation of the remarkable role played by structural effects in molecular evolution. 

In conclusion, complex mixtures undergo processes subjected to Molecular Darwinism. This term is particularly useful for summarizing what happens at the borderline between prebiotic Chemistry and rudimental pre-Biology, possibly opening up the clarification of relevant mechanisms and interactions. From our privileged point of observation of living beings, we may a posteriori add Chemomimesis as an analytical tool to indirectly reconstruct these phenomena.
